# Comparative Genomic Analysis of the Foodborne Pathogen *Burkholderia gladioli* pv. *cocovenenans* Harboring a Bongkrekic Acid Biosynthesis Gene Cluster

**DOI:** 10.3389/fmicb.2021.628538

**Published:** 2021-05-17

**Authors:** Zixin Peng, Tania Dottorini, Yue Hu, Menghan Li, Shaofei Yan, Séamus Fanning, Michelle Baker, Jin Xu, Fengqin Li

**Affiliations:** ^1^NHC Key Laboratory of Food Safety Risk Assessment, Chinese Academy of Medical Sciences Research Unit (2019RU014), China National Center for Food Safety Risk Assessment, Beijing, China; ^2^School of Veterinary Medicine and Science, University of Nottingham, Sutton Bonington Campus, Leicestershire, United Kingdom; ^3^UCD-Centre for Food Safety, School of Public Health, Physiotherapy and Sports Science, University College Dublin, Dublin, Ireland

**Keywords:** *Burkholderia gladioli* pv. *cocovenenans*, bongkrekic acid, food-borne poisoning, bongkrekic acid biosynthesis gene cluster, recombination, virulence, toxin

## Abstract

The environmental bacterium *Burkholderia gladioli* pv. *cocovenenans* (*B. cocovenenans*) has been linked to fatal food poisoning cases in Asia and Africa. Bongkrekic acid (BA), a mitochondrial toxin produced by *B. cocovenenans*, is thought to be responsible for these outbreaks. While there are over 80 species in the *Burkholderia* genus, *B. cocovenenans* is the only pathovar capable of producing BA and causing human death. However, the genomic features of *B. gladioli* and the evolution of the BA biosynthesis gene cluster, *bon*, in *B. cocovenenans* remain elusive. In this study, 239 whole genome sequences (WGSs) of *B. gladioli*, isolated from 12 countries collected over 100 years, were used to analyze the intra-species genomic diversity and phylogenetic relationships of *B. gladioli* and to explore the origin and evolution of the *bon* gene cluster. Our results showed that the genome-wide average nucleotide identity (ANI) values were above 97.29% for pairs of *B. gladioli* genomes. Thirty-six of the 239 (15.06%) *B. gladioli* genomes, isolated from corn, rice, fruits, soil, and patients from Asia, Europe, North America, and South America, contained the *bon* gene cluster and formed three clades within the phylogenetic tree. Pan- and core-genome analysis suggested that the BA biosynthesis genes were recently acquired. Comparative genome analysis of the *bon* gene cluster showed that complex recombination events contributed to this toxin biosynthesis gene cluster’s evolution and formation. This study suggests that a better understanding of the genomic diversity and evolution of this lethal foodborne pathovar will potentially contribute to *B. cocovenenans* food poisoning outbreak prevention.

## Introduction

*Burkholderia gladioli* is ubiquitous in soil and plants (Eberl and Vandamme, 2016). Some pathogenic strains of this species can be subdivided into four pathovars: *B. gladioli* pv. *agaricicola*, *B. gladioli* pv. *alliicola*, *B. gladioli* pv. *gladioli*, and *B. gladioli* pv. *cocovenenans* (*B. cocovenenans*, also called *Pseudomonas cocovenenans* in some previous literature) (Lee et al., 2016). In contrast to the first three plant pathovars, *B. cocovenenans* can cause lethal food poisoning by producing a highly unsaturated tricarboxylic fatty acid, bongkrekic acid (BA), which is a mitochondrial toxin that can efficiently block the mitochondrial adenine nucleotide translocator (ANT) (Moebius et al., 2012; Anwar et al., 2017). BA is also called a respiratory toxin since this toxin can prevent the respiratory chain phosphorylation (Rohm et al., 2010). BA causes food poisoning with an acute toxicity of 1.41 mg/kg (LD_50_ by intravenous injection on mice) (Fujita et al., 2018). In epidemiological investigations, *B. cocovenenans* and BA were detected simultaneously in coconut- and corn-based products responsible for food-borne outbreaks in Indonesia, China, and Mozambique (Anwar et al., 2017; Gudo et al., 2018; Li J. et al., 2019). A dose–response relationship has been found between the amount of BA-contaminated food consumed and illness severity, with reported manifestations of BA poisoning including abdominal pain, diarrhea, vomiting, weakness, and palpitations (Gudo et al., 2018).

As an important fatal toxin, BA is an odorless, tasteless, heat-stable substance, and contaminated food matrices can have a normal appearance, smell, and taste. Thus, BA can be difficult to detect during the food preparation and consumption process (Gong et al., 2016; Anwar et al., 2017). The compound is not expected to volatilize or hydrolyze in the environment, but may be susceptible to direct photolysis by sunlight (Voragen et al., 1982). It is important to note that cooking foods contaminated with BA does not render them safe for consumption: although the bacteria are destroyed, the toxin itself is heat-stable (Falconer et al., 2017). Previous literature indicated that food poisoning events involving BA were found only in specific regions of Indonesia and China, as a result of consuming locally produced fermented foods, but posed a massive health threat and caused many lethal intoxications (Matsumoto et al., 2015; Falconer et al., 2017). Outbreaks of BA food poisoning in these two regions exhibit high mortality rates, with numbers of 40% and 60% being reported on China and Indonesia, respectively (Anwar et al., 2017).

Besides BA, *B. cocovenenans* also produces another toxin, toxoflavin (TF), an electron carrier that generates hydrogen peroxide and subsequent toxicity related to free radical formation (Meng et al., 1988a,b; Falconer et al., 2017; Li X. et al., 2019). However, TF toxicity is relatively mild and secondary to that of BA. BA is well recognized to account for the lethal food poisoning incidents (Lee et al., 2016; Anwar et al., 2017).

The most recent reported lethal food poisoning case caused nine people’s deaths in Heilongjiang Province of China in October 2020, after consuming a homemade sour soup made of fermented corn, with a case fatality rate of 100% (Yuan et al., 2020). An earlier BA poisoning outbreak case leading to five people deaths, was recorded in Guangdong Province of China in 2018, and was caused by consuming a commercially produced rice noodle product which was not fermented or noticeably spoiled (Li J. et al., 2019). This clinical case report showed that non-fermented food could also lead to BA food poisoning. Notably, the first report of BA food poisoning occurred outside Asia was severe. In January 2015, 75 people died and 177 were hospitalized after consuming a traditional beverage made from corn flour in Mozambique, Africa (Gudo et al., 2018). This tragic event illustrates that *B. cocovenenans* and its toxic product BA are of global concern.

The *Burkholderia* genus has more than 80 species, including the notorious human pathogens *B. mallei* and *B. pseudomallei* and some opportunistic human pathogens, but *B. cocovenenans* is the only human pathovar found to produce BA and linked to food poisoning deaths (Depoorter et al., 2016; Anwar et al., 2017). The genetic basis for BA biosynthesis in *B. cocovenenans* depends on harboring the *bon* gene cluster, which encodes a *trans*-AT subgroup of modular type I polyketide synthase (Type I PKS) and accessory enzymes catalyzing complex polyunsaturated tricarboxylic acid assembly (Moebius et al., 2012). Type I PKS are typically encoded in close genomic vicinity and organized in gene clusters. This type of enzyme is defined as multidomain with a modular synthetic scheme, in which each module typically has a minimal set of three core domains, namely acyltransferase (AT), acyl carrier protein (ACP), and ketosynthase (KS), and is responsible for the incorporation of a single building block of acyl-CoA (Wang et al., 2015). The evolutionary origins of the complex PKS biosynthetic pathways remain to be determined.

Previous studies on *B. cocovenenans* and BA have mostly focused on clinical case reports (Falconer et al., 2017; Shi et al., 2019), BA synthesis (Rohm et al., 2010; Moebius et al., 2012; Matsumoto et al., 2015; Fujita et al., 2018), and apoptosis inhibition (Matsumoto et al., 2015; Takeda et al., 2016; Kano et al., 2017, 2019). However, the origin and evolution of BA biosynthesis gene cluster, *bon*, in *B. cocovenenans* remain to be elucidated. In this study, we performed whole genome sequencing and determined the evolutionary origins of the *bon* gene cluster in pathovar *B. cocovenenans* Co14, as well as in other *B. gladioli* strains containing this gene cluster. We conducted a genome-wide comparison of *B. cocovenenans* Co14 against other *B. gladioli* genomes to provide deeper insights into the genomic diversity and the evolutionary origins of the *bon* gene cluster in this lethal foodborne pathogen. We showed evidence for recombination events occurring in the BA biosynthesis gene cluster evolution in *B. cocovenenans*.

## Materials and Methods

### Bacterial Strain

In 1977, four people from one family died following the consumption of contaminated fermented corn flour in Tonghe County, Heilongjiang Province, China. A *B. cocovenenans* strain (named *B. cocovenenans* Co14) and its toxic product BA were identified from the remaining food and linked as the etiological agent in this event. The serotype of *B. cocovenenans* Co14 was identified as O-IV (Meng et al., 1988a). This strain was the first isolated and identified as a BA-producing pathogen causing fermented corn flour poisoning in China (Meng et al., 1988b). It was stored at −80°C in the Microbiology Laboratory of the China National Center for Food Safety Risk Assessment.

### Genome Sequencing, Assembly and Annotation

*Burkholderia cocovenenans* Co14 was subjected to genomic DNA extraction using the E.Z.N.A.^®^ Bacterial DNA Kit (Omega Bio-Tek, Norcross, GA, United States), in accordance with the manufacturer’s protocol. *B. cocovenenans* Co14 underwent genomic sequencing on both a Pacific Biosciences RS II sequencing platform (Pacific Biosciences, Menlo Park, CA, United States) and an Illumina Hiseq 2500 PE150 platform (Illumina, San Diego, CA, United States). Briefly, single-molecule real-time (SMRT^®^) sequencing was conducted using the C4 sequencing chemistry and P6 polymerase with one SMRT^®^ cell. For Illumina sequencing, the template genomic DNA was fragmented by sonication to a size of 350 bp using NEBNext^®^ Ultra^TM^ DNA Library Prep Kit for Illumina (NEB, United States) and sequenced. The PacBio and Illumina sequencing reads were assembled *de novo* using a hybrid assembly algorithm implemented in Allpaths-LG software^1^ (v44620) (Shibata et al., 2013). The complete genome sequence of *B. cocovenenans* Co14 was deposited in GenBank under the accession number CP033430.1 (chromosome 1), CP033431.1 (chromosome 2) and CP033429.1 (plasmid pCO1).

In total, 239 un-contaminated *B. gladioli* genomes and their genome information were downloaded from the NCBI Genome Database^2^ and PATRIC 3.6.5^3^. Characteristics of the single isolates, including collection details, genome assembly statistics, genomic features, origins etc are detailed in Supplementary Dataset 1. Prokka v.1.14.0 (Seemann, 2014) was used to annotate the 239 assembled genomes, including the *B. cocovenenans* Co14 genome. In addition, 4080 genus *Burkholderia* genomes and their information (Supplementary Dataset 2) were also download from the PATRIC Database.

### Average Nucleotide Identity (ANI) Analysis

Average nucleotide identity values were calculated between the genomes of *B. cocovenenans* Co14 and the other 238 *B. gladioli* strains using FastANI v1.32^4^ (Jain et al., 2018). The pheatmap package in R (v4.0.0) (R Core Team, 2020) was used to perform hierarchical clustering and visualization^5^. FastANI results for each genome pair was transformed to an ANI matrix. Function *pheatmap* was applied with clustering distance method “Euclidean” and the clustering method “complete.”

### Core- and Pan-Genome and Phylogenetic Analyses

The core- and pan-genome of the *B. gladioli* genomes were estimated using Roary v3.12.0^6^ (Page et al., 2015). A 95% identity cutoff was used, and core genes were defined as those contained in 99% of the genomes analyzed (≥236). Roary provided three figures using the roary_plots.py script^7^, which summarized the output: a graph showing how the number of genes changed in the core/pan genome; a pie chart summarizing the conservation of genes and the number of genomes where they were present; a figure showing the presence and absence of core and accessory genes. A maximum-likelihood phylogenetic tree was constructed with the single nucleotide polymorphisms (SNPs) of the core gene alignment using FastTree 2.1 with the Jukes-Cantor + CAT nucleotide substitution model (Price et al., 2010). The core SNPs were extracted using snp-sites v2.5.1^8^ (Keane et al., 2016). The phylogenetic tree was visualized with the gene presence/absence. The *B. glumae* ASM993137 (accession number: GCA_009931375.1) sequence was used as an outgroup for phylogenetic tree rooting.

### BA Biosynthetic Gene Cluster *bon* Analysis

The 67.5-kbp BA biosynthesis gene cluster *bon* (GenBank Accession JX173632) from *B. cocovenenans* DMSZ11318 was used as the reference sequence. BLASTn+ (v. 2.9.0) was used to align the *B. cocovenenans* Co14, 239 *B. gladioli* and 4080 *Burkholderia* spp. genomes individually to the reference sequence, with a minimum percentage identity of 90%. The open reading frames (ORFs) of the *bon* gene cluster in the 36 *B. gladioli* genomes were annotated according to the previously reported BA biosynthesis gene cluster of *B. cocovenenans* DMSZ11318 and *B. gladioli* BSR3 (Moebius et al., 2012). Also, the BLAST tool from NCBI aligned the ORFs of the *bon* gene cluster of *B. cocovenenans* Co14 and each coding ORF sequence in the Non-redundant protein sequences (nr) Database to search the most highly related proteins in non-*B*. *gladioli* species. Easyfig^9^ (Sullivan et al., 2011) was used to create a linear comparison figure depicting the *bon* gene clusters of *B. gladioli* along with homologous flanking sequences identified from GenBank. antiSMASH 5.0^10^ (Blin et al., 2019) was also used to identify, annotate, and analyze the *bon* gene cluster in the *B. cocovenenans* Co14 genome using the “relaxed” detection strictness mode.

### Genomic Island (GI) Analysis

Three GI prediction methods, IslandPick, SIGI-HMM, and IslandPath-DIMOB, integrated by IslandViewer 4 (Bertelli et al., 2017) were used as guides for the identification of GIs in the three complete *bon* gene cluster-containing chromosomes *B. cocovenenans* Co14, *B. gladioli* BSR3, and *B. gladioli* 3723STDY6437373. The location of the *bon* gene cluster of *B. cocovenenans* Co14, *B. gladioli* BSR3, and *B. gladioli* 3723STDY6437373 were compared with the predicted GI.

## Results

### Features of the *B. cocovenenans* Co14 Genome

The complete genome sequence of *B. cocovenenans* Co14 consisted of two independent closed circular chromosomes and one circular plasmid. Chromosome 1 contained 4,171,651 bp with 3,729 protein-coding ORFs, while chromosome 2 contained 4,002,946 bp with 3,290 protein-coding ORFs. The GC content of chromosomes 1 and 2 were 67.82% and 68.32%, respectively. Both showed vague GC skew at their origins of replication. The size of the plasmid pCO1 was found to be 145,928 bp with a GC content of 63.25%, encoding 139 ORFs.

### Distribution and Genome Characteristics of *B. gladioli*

*Burkholderia gladioli* are widely distributed and commonly found in different environments (Supplementary Dataset 1). The 239 strains included in this study were isolated throughout the world, including North America (United States and Canada), South America (Brazil and Colombia), Asia (China, South Korea, Indonesia, and India), Europe (United Kingdom and Italy) and Africa (Zimbabwe). The origin of these strains included plants, such as corn, rice, gladiolus and onion; human clinical samples, including cystic fibrosis, sputum, nose-ethmoid sinus and ethmoid sinus; animal samples including beetle eggs; and environment samples including water and soil. The most studied strain *B. gladioli* ATCC^®^ 10248 was originally isolated from *Gladiolus* sp. in the United States in 1913. Seven of the 239 strains had complete whole genome sequences published in the NCBI Genome Database. All possessed 2 chromosomes and 1–4 plasmids. The sizes of the 239 genomes were between 7.33–9.31 Mbp (Mean: 8.31 Mbp; Median: 8.32 Mbp) with GC contents of 67.34%–68.30% (Mean: 68.05%; Median: 68.21%).

### ANI Analysis of *B. gladioli*

**FIGURE 1:**
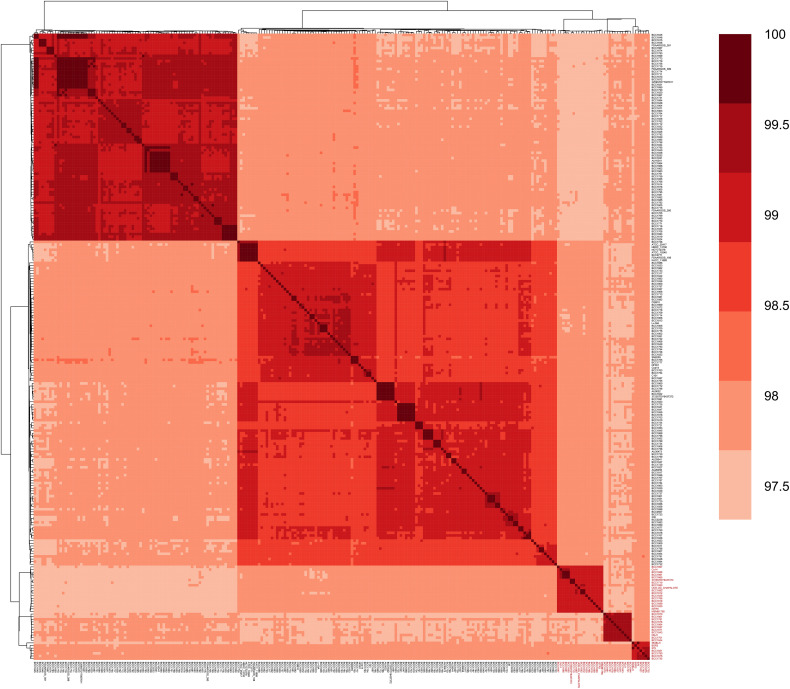
Heatmap of the average nucleotide identity (ANI) of *Burkholderia gladioli* genomes. Pairwise comparison was applied between 239 genomes. The 36 *B. gladioli* genomes containing the BA biosynthesis gene cluster *bon* are shown in red color and bold text. *B. cocovenenans* Co14 forms a cluster with *B. gladioli* BCC1661, *B. gladioli* BCC1686 and *B. gladioli* BCC1665 genomes with an alignment fraction over 99.41%, while *B. gladioli* ISTR5 and *B. gladioli* BCC1829 showed an identity of 99.02% and 98.95%, respectively.

To assess similarity among *B. gladioli* genomes, ANI values were calculated, and a clustered heatmap was generated. As shown in Figure 1, all *B. gladioli* genomes exhibited pairwise ANI values of 97.29%∼100.00% (mean 98.25%), above the 95% suggested cut-off for species identification (Goris et al., 2007; Jain et al., 2018).

*Burkholderia cocovenenans* Co14 shared the greatest ANI (99.42%) with *B. gladioli* BCC1661 (isolated from a clinical sample, United States), followed by *B. gladioli* BCC1686 and BCC1665 (both isolated from clinical samples, United States) and *B. gladioli* UCD-UG_CHAPALOTE (isolated from chapalote corn, Canada, 2008). In contrast, *B. cocovenenans* Co14 only shared 97.42% and 97.47% identity with *B. gladioli* BCC1828 and *B. gladioli* BCC1684 (isolated from clinical samples from Canada and United States, respectively). A previously reported BA biosynthesis gene cluster-containing strain *B. gladioli* BSR3, which was isolated from a rice sheath in South Korea in 2011, was most similar to *B. gladioli* 579, a strain isolated from mature oil palm fruits in Brazil in 2015, with an ANI value of 99.16%.

### Core- and Pan-Genome of *B. gladioli*

**FIGURE 2:**
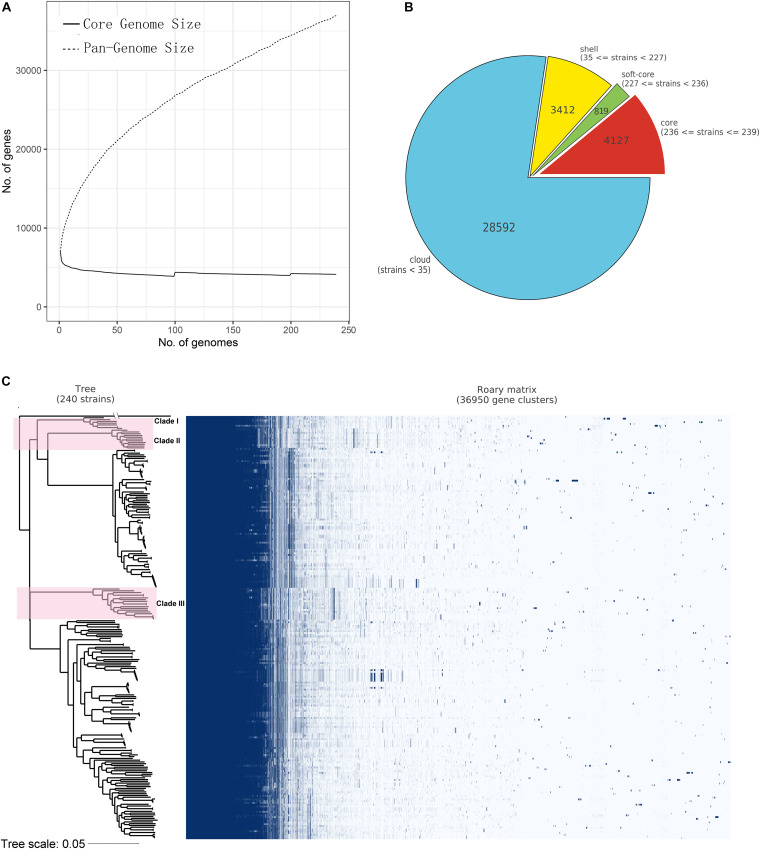
Core- and pan-genome analysis of *Burkholderia gladioli* based on 239 genomes. **(A)** Core- and pan-genome profile curves of *B. gladioli* reporting how the core- and pan- genes vary as genomes are added in a random order. The solid line and dotted line denote the size of the core- and pan-genome size of *B. gladioli*, as well as the relationship between core- and pan-genome size and genome number, respectively. On the *y* axes, the number of genes is reported, while on the *x* axes, the number of strains considered is shown. **(B)** The pie chart illustrates the number of genes belonging to the core, the soft core, the shell, or the cloud of the *B. gladioli* species. **(C)** Core and accessory genes and the core-genome SNPs phylogenetic relationship of *B. gladioli*. The left tree was constructed based on the core-genome SNPs of *B. gladioli* species using a maximum-likelihood method, and the right matrix plot denotes the presence and absence of every gene over all strains. The 36 strains containing the bongkrekic acid biosynthetic gene cluster *bon* are formed three clades and shown in pink background color on the tree. The *B. glumae* ASM993137 genome was used as an outgroup. The tree scale was shown in the bottom.

Insights into the pan- and core-genome properties of all 239 *B. gladioli* sequences were obtained using Roary (Figure 2). A total of 36,950 genes were identified in the pan-genome of *B. gladioli*, 4,127 of which were considered to be core genes (present in ≥99% of strains, Figure 2A). The numbers of soft-core genes (95% ≤ strains < 99%), shell genes (15% ≤ strains < 95%) and cloud genes (0 ≤ strains < 15%) were 819, 3,412 and 28,592, respectively (Figure 2B).

A maximum-likelihood phylogenetic tree was constructed based on the core genome SNPs of *B. gladioli* isolates (Figure 2C and Supplementary Figure 1). As shown in Supplementary Figure 1 and Supplementary Dataset 1, *B. gladioli* isolated from human clinical samples, food, plants, animals and environmental sources were distributed across multiple clusters. *B. cocovenenans* Co14 had the closest phylogenetic relationship with *B. gladioli* BCC1686, followed by *B. gladioli* BCC1661, *B. gladioli* BCC1665, and *B. gladioli* UCD-UG_CHAPALOTE, similar to the ANI results. *B. gladioli* BSR3 was found to have close phylogenetic relationships with *B. gladioli* BCC1735, *B. gladioli* BCC1780, and *B. gladioli* BCC1675.

### BA Biosynthesis Gene Cluster Analysis

Based on the results of BLASTn+, a 66,726-bp sequence (located between positions 2,961,077∼3,027,802 bp in the genome) was found in chromosome 1 of *B. cocovenenans* Co14, with an identity of 99.39% to the BA biosynthesis gene clusters *bon* of *B. cocovenenans* DMSZ11318 (67,546-bp). In addition, this *bon* gene cluster of *B. cocovenenans* DMSZ11318 was also found in 36 genomes of *B. gladioli* with an identity range of 97.93%–99.95%, respectively (Supplementary Dataset 3 and Supplementary Table 1). Twenty-six of the 36 *B. gladioli* genomes (72.2%) originated from human clinical samples from the United States. In addition, among the 4080 *Burkholderia* spp. genomes (Supplementary Dataset 2), the BA biosynthesis gene cluster *bon* was found only in the above 36 *B. gladioli* genomes.

Interestingly, the 36 genomes containing the BA biosynthesis gene cluster formed three clusters in the ANI heatmap (Figure 1) and three clades in the core SNPs phylogenetic tree (Figure 2C and Supplementary Figure 1). The ANI range of the 36 BA biosynthesis gene cluster-containing *B. gladioli* genomes was 97.45%∼100.00% with an average value of 98.28%, slightly higher than the average value of 329 genomes (98.25%). The average identity of the 36 *bon* gene clusters was 98.53%, which was higher than the average genome-wide ANI value (98.28%) of the 36 *bon* gene cluster-containing genomes. These 36 genomes contain the intact BA biosynthesis gene cluster *bon* (Table 1 and Supplementary Dataset 4). The *bon* gene cluster-containing *B. cocovenenans* DMSZ11318 and *B. cocovenenans* Co14 were proved to produce BA by experiment. This suggested that these 36 genomes are likely to produce BA. As such, we theorize that these strains can produce BA and are able to cause food poisoning. However, at the present experimental work on these strains is lacking, and it remains unknown whether they can produce BA or cause any human foodborne disease.

**TABLE 1:** Comparison of the bongkrekic acid biosynthesis gene clusters of *B. cocovenenans* DMSZ11318, *B. cocovenenans* Co14, *B. gladioli* BSR3, *B. gladioli* 3723STDY6437373, *B. gladioli* MSMB1756, *B. gladioli* BCC1650, and *B. gladioli* BCC1837.

*B. cocovenenans* DMSZ11318				*B. cocovenenans* Co14			*B. gladioli* BSR3		*B. gladioli* 3723STDY6437373		*B. gladioli* MSMB1756		*B. gladioli* BCC1650		*B. gladioli* BCC1837	
						
Protein	Size (aa)	Predicted function^#^	Accession number	Size (aa)	Role in BA biosynthesis*	Present/absent (±)	Size (aa)	Present/absent (±)	Size (aa)	Present/absent (±)	Size (aa)	Present/absent (±)	Size (aa)	Present/absent (±)	Size (aa)	Present/absent (±)
BonL	417	Cytochrome P450 monooxygenase	AFN27475	417	Addition	+	410	+	417	+	417	+	417	+	417	+
BonJ	337	Acyl transferase	AFN27476	337	Core	+	337	+	337	+	337	+	337	+	337	+
BonK	377	Acyl transferase	AFN27477	377	Core	+	377	+	377	+	377	+	377	+	377	+
BonF	416	KS (involved in β-branching)	AFN27478	416	Core	+	416	+	416	+	416	+	416	+	416	+
BonG	424	3-Hydroxy-3-methylglutaryl-CoA synthase	AFN27479	424	Core	+	419	+	419	+	419	+	424	+	424	+
BonA	7908	Polyketide synthase	AFN27480	8093	Core	+	8130	+	8122	+	8121	+	6805	+	8112	+
BonB	3582	Polyketide synthase	AFN27481	5303	Core	+	5299	+	5297	+	5297	+	5301	+	5297	+
BonC ^†^	1705	Polyketide synthase	AFN27482	–	–	–	–	–	–	–	–	–	–	–	–	–
BonD	4096	Polyketide synthase	AFN27483	3986	Core	+	4072	+	4099	+	3602	+	4103	+	3995	+
BonE	450	Enoyl reductase	AFN27484	450	Addition	+	450	+	450	+	450	+	450	+	450	+
BonH^¥^	276	Enoyl-CoA hydratase		272	Addition	+	272	+	272	+	272	+	276	+	272	+
BonI	249	Enoyl-CoA hydratase	AFN27485	249	Addition	+	249	+	249	+	249	+	249	+	249	+
BonM	269	*O*-methyltransferase	AFN27486	269	Addition	+	269	+	269	+	269	+	269	+	269	+

^#^The predicted function referred to the work of Moebius et al. (2012). *The protein role in BA biosynthesis was predicted by antiSMASH 5.0 (https://antismash.secondarymetabolites.org/). ^†^The polyketide synthase was encoded on four ORFs BonABCD in *B. cocovenenans* DMSZ11318 since single nucleotide substitutions made a stop codon in *bonB*, in contrast with that of only three ORFs BonABD in other *bon* gene cluster-containing genome. ^¥^BonH of *B. cocovenenans* DMSZ11318 was missing by annotation error.

### ORFs of BA Biosynthesis Gene Cluster *bon*

**FIGURE 3:**
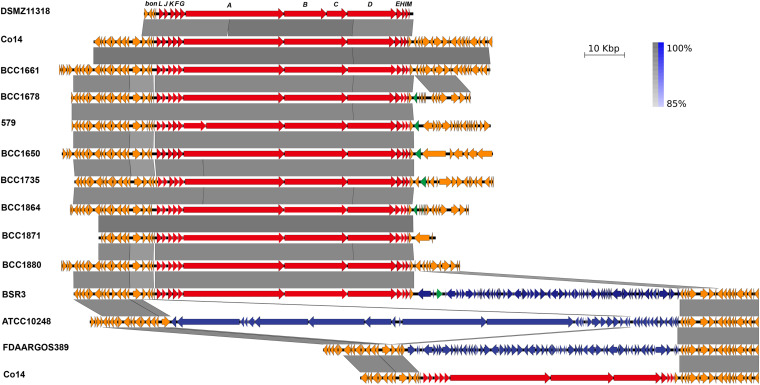
Linear alignment and detailed structure of the 11 typical bongkrekic acid (BA) biosynthetic gene cluster *bon* and its flanking homologous sequences. The gene product names are labeled on the top of *B. cocovenenans* DMSZ11318 *bon* gene cluster. The red arrows represented the ORF*s* in the *bon* gene cluster, the orange arrows represent the flanking sequences, the blue arrows represent non-*bon* genes between the flanking homologous sequences, and the green arrows represent the transposase encoding sequences.

As shown in Table 1 and Figure 3, 12 ORFs designated *bonLJKFGABDEHIM* in *B. cocovenenans* Co14 were annotated as being involved in BA biosynthesis. The ORFs in the *bon* gene cluster of *B. cocovenenans* Co14 that were present or absent in *B. cocovenenans* DMSZ11318, as well as the 35 other *B. gladioli* genomes, are shown in Table 1 and Supplementary Dataset 4. Further analysis of the ORFs of the *bon* gene cluster in the *B. gladioli* core- and pan-genomes showed that the 12 genes *bonLJKFGABDEHIM* belonged to shell genes of pan-genome.

All the *bon* gene clusters displayed greater synteny with *B. cocovenenans* Co14 than with *B. cocovenenans* DMSZ1131, although from PKS assembly lines, only three ORFs (BonABD) are encoded on *B. cocovenenans* Co14 while four ORFs (BonABCD) are on *B. cocovenenans* DMSZ1131 (Figure 3 and Supplementary Dataset 4). The differences in the amino acid sequences found in PKS assembly lines were primarily due to single nucleotide substitutions that are more prominent in the PKS part of the *bon* gene cluster of *B. cocovenenans* DMSZ11318, and made a stop codon in *bonD*. Similarly, a single nucleotide substitution is present in the *bonA* of *B. gladioli* 579 and formed two ORFs in this gene.

The antiSMASH program facilitates rapid genome-wide identification, annotation and analysis of secondary metabolite biosynthesis gene clusters in bacterial and fungal genomes. A *trans*-AT PKS responsible for BA production was identified in this region of the *B. cocovenenans* Co14 chromosome 1. The genes *bonJKFGABD* were identified as the core BA biosynthetic genes, while *bonLEHIM* were identified as additional biosynthetic genes (Table 1).

### Genetic Recombination Identification

By aligning the two flanking homologous sequences of the *B. cocovenenans* Co14 *bon* gene cluster with other *bon* gene cluster-containing or non-containing *B. gladioli* genomes, it was found that the down flanking sequence showed more diversity than that of the upper flanking sequence (Figure 3 and Supplementary Figure 2). The sequence between both homologous flanking sequences may represent a gene recombination hotspot. The two flanking homologous sequences of the *B. cocovenenans* Co14 *bon* gene cluster were conserved in *B. gladioli*, but additional sequences were found to be inserted between them. A 67,680-bp locus encoding 65 ORFs was inserted downstream of the *bon* gene cluster in *B. gladioli* BSR3. Most of the inserted encoding genes were of unknown function. A high identity gene sequence was also found in *B. gladioli* FDAARGOS_389, a strain originating from an onion, but the *bon* gene cluster was absent upstream of this sequence. However, in *B. gladioli* ATCC_10248 and *B. gladioli* KACC_11889, a 128-kbp high-identity sequence was inserted between the two flanking homologous sequences and encoded 35 ORFs. As in *B. gladioli* BSR3, most encoding genes in this region were of unknown function.

Notably, in some genomes of *bon*-containing *B. gladioli* (BCC1650, BCC1675, BCC1678 et al.), adjacent to the *bonM* gene, *IS*NCY family transposase *IS*Bcen27 was found, while in the genome of *B. gladioli* BCC1870, it was *IS*3 family transposase *IS*Bp1 instead. It showed that this cluster may have been acquired by horizontal gene transfer (HGT).

Among the 36 BA biosynthesis gene cluster-containing *B. gladioli* genomes, the *B. cocovenenans* Co14, *B. gladioli* BSR3, and *B. gladioli* 3723STDY6437373 genome were closed. As shown in Supplementary Figure 3, the *bon* gene cluster was located within one large GI (located between positions 2,962,767∼3,025,227 bp) of *B. cocovenenans* Co14 chromosome 1 which was identified by IslandPick (Langille et al., 2008). This region included the ORFs *bonLJKFGABDE*. Interestingly, the *bon* gene cluster was located within one large GI (2,430,789∼2,496,466 bp) in one chromosome of *B. gladioli* 3723STDY6437373. This region included the ORFs *bonLJKFGABDEHIM*. In contrast, the *bon* gene cluster was not located in any GI in *B. gladioli* BSR3 chromosome 1.

### Origin Analysis of the BA Biosynthesis Gene Cluster

A search using the complete *bon* gene cluster of *B. cocovenenans* Co14 with BLASTn showed that this sequence region had a high query coverage and identity with that found in the 36 BA biosynthesis gene cluster containing *B. gladioli* genomes (Table 2 and Supplementary Table 2). ORFs found in non-*B. gladioli* species (BonG, BonE, BonI and BonM) showed higher query coverage (>90%) and identities (>70%) with proteins from *Pseudomonas* sp. R26(2017), *Podila verticillate* CPC16_007748 and *Trinickia diaoshuihuensis*, which showed that these genes may originate from non-*B. gladioli* species or inverse (Table 2). By contrast, BonL, BonJ, BonK, BonF, BonA, BonB, BonD, and BonH showed lower identities with proteins from other non-*B. gladioli* species, which may show that these genes may originate via an intra-species inheritance pathway.

**TABLE 2:** Closest relative protein in non-*Burkholderia gladioli* species of the core and additional bongkrekic acid biosynthesis genes.

Product	Role in BA biosynthesis	Closest relative of non-*B. gladioli* species (sequence ID)	Query cover (%)	Identity (%)
BonL	Addition	*Acidobacteria bacterium* 13_2_20CM_56_17 (OLB28637.1)	97	46.94
BonJ	Core	*Burkholderia cepacia* (WP_105393823.1)	91	54.87
BonK	Core	*Pseudomonas* sp. MSSRFD41 (WP_185699837.1)	100	60.16
BonF	Core	*Pseudomonas aestus* P308_15900 (ERO59990.1)	97	69.14
BonG	Core	*Pseudomonas* sp. R26(2017) (WP_085653298.1)	98	77.43
BonA	Core	*Methylomusa anaerophila* (WP_126310258.1)	91	47.65
BonB	Core	*Pseudomonas acidophila* BWP39_00030 (PCE28611.1)	85	50.41
BonD	Core	*Chitinophaga pinensis* (WP_012792895.1)	98	42.35
BonE	Addition	*Podila verticillate* CPC16_007748 (KAF9395587.1)	93	70.85
BonH	Addition	*Candidatus Desulfosporosinus infrequens* (WP_106800322.1)	93	58.43
BonI	Addition	*Trinickia diaoshuihuensis* (WP_116137735.1)	97	72.84
BonM	Addition	*Trinickia diaoshuihuensis* (WP_116137737.1)	99	72.01

## Discussion

*Burkholderia* species are incredibly diverse and versatile bacteria that possess large multi-replicon chromosomes along with several large plasmids (>100 kbp), which enable these bacteria survive different environmental conditions (De Felice et al., 2016; Mullins et al., 2020). The majority of life essential genes in multiple-chromosome bacteria are usually located on one primary chromosome, with additional chromosomes containing fewer essential genes, being mainly composed of niche-specific genes (Losada et al., 2010). In the case of *Burkholderia* species, most genes necessary for the basic life processes were located on chromosome 1 (Spring-Pearson et al., 2015).

*Burkholderia* species are also known as prolific producers of secondary metabolites with potent biological and pharmacological properties (Ross et al., 2014; Mullins et al., 2020). BA is an important secondary metabolite produced by *B. cocovenenans*, a pathovar belonging to *B. gladioli* (Ross et al., 2014). Besides being recognized as a harmful toxin, BA is also associated with inhibiting apoptosis and can be applied to protect neuronal death in the cortex (Okazaki et al., 2015). BA plays this role in coordinated (apoptosis) and uncoordinated (necrotic) cell death by inhibiting the mitochondrial permeability transition pore (MPTP) via binding to the ANT in mitochondria (Anwar et al., 2017). This compound is synthesized by a multi-modular type I PKS (also called *trans*-AT PKS) along with accessory enzymes that function to catalyze assembly the polyunsaturated tricarboxylic acid (Moebius et al., 2012).

Another intensive studied *Burkholderia* toxin, TF, revealed that both regulatory genes and biosynthesis genes were important for toxin production (Seo et al., 2015; Lee et al., 2016). Moebius et al. (2012) predicted that two putative regulatory genes *bonR1-R2* were located upstream of the *bon* genes. However, the function of these two genes have not been proven experimentally. In contrast with TF, which were produced by both *B. gladioli* and *B. glumae*, BA biosynthesis gene clusters were only found in some *B. gladioli* genomes in this study.

Previous phylogenetic studies indicated that *Burkholderia* species constituted two distinct lineages: the larger cluster included mainly plant growth-promoting bacteria and the other cluster was dominated by human, animal and plant pathogens (Baldeweg et al., 2019). In our study, *B. gladioli* strains originating from human, plant and environmental sources were distributed across multiple clusters. Moreover, the *bon* gene cluster-containing *B. gladioli* strains of human, plant, food, and environmental origins from different countries were found to have a close phylogenetic relationship. The 36 *B. gladioli* strains formed three clades on the phylogenetic tree using core SNPs. This may be caused by that the toxin gene cluster was acquired from a common ancestor *B. gladioli* species, and then inherited vertically, or that this gene cluster was gained in specific *B. gladioli* genomes via HGT. Apart from the gain of this gene cluster, it may also be possible that the ancestral gene losses of this cluster during the inheritance resulted in the absence of the other strains.

Pangenomic diversity in *B. gladioli* was high and their pangenomes were “*open*” as revealed by core- and pan-genome analysis. The core genes of *B. gladioli* comprised 11.17% of the complete set of genes and this proportion would likely reduce with a greater number of samples. However, though the genomes of *B. gladioli* have large diverse pan-genome, our results also revealed high ANI values shared by *B. gladioli*, far above the 95% intra-species cutoff value (Jain et al., 2018). Interestingly, the BA biosynthesis genes of *B. gladioli* were all defined as shell genes, which are those genes often present but lacking in subsets of genomes and also thought to be more recently acquired (Deschamps et al., 2014; Snipen and Liland, 2015).

Surprisingly, 36 of the 329 published *B. gladioli* genomes, isolated from food, plants, soil and patients globally from 1977 to 2014, contained the BA biosynthesis *bon* gene cluster, which implicated that the prevalence of this foodborne pathogen was wider than that of only in Asia and Africa, as previously thought. Notably, the United States did not report any food poisoning caused by *bon-*containing *B. gladioli* strains, though several such strains were isolated. However, *B. cocovenenans*-food poisoning outbreaks have only been reported in China, Indonesia and Africa which may be attributed to the following: (1) Region-specific food products. The consumption of some local traditional food, such as tempe bongkrek in Indonesia, fermented corn products and *Tremella fuciformis* mushrooms in China and fermented corn flour-based beverages in Mozambique, which were contaminated by *B. cocovenenans* and BA, caused food poisoning and death. (2) Sanitary conditions. BA is produced in warm environments (22∼30°C) with a neutral pH. The presence of fatty acids in coconut and corn will promote the production of BA in *B. cocovenenans*. High-risk foods are more likely to be contaminated by *B. cocovenenans* under unsanitary conditions. (3) Under-reporting. A lack of confirmatory testing capacity for detection of *B. cocovenenans* or BA or a failure to consider the diagnosis could be contributing to misdiagnosis in other parts of the world, as *B. cocovenenans* is ubiquitous in plants and soil (Anwar et al., 2017).

Recombination plays an important role in the evolution of niche-specific gene pools thereby facilitating genome flexibly in the ecological speciation of bacteria. Recombination events can be classically divided into different types: homologous *versus* non-homologous or illegitimate recombination, the latter often being termed HGT (Mostowy et al., 2017). In *B. gladioli*, some transposases were found adjacently to *bon* clusters, which suggested that both recombination and HGT events may have affected to this cluster formation and spread.

Genomic islands are genomic regions that can serve as a driving force to virulence. They may contain a few or many genes which can be acquired through recombination from other bacteria (Patil et al., 2017). In *B. pseudomallei*, GIs were a key feature of the genome, accounting for a major source of genomic diversity, as well as being associated with pathogenicity in humans (Sim et al., 2008). High rates of gene transfer and recombination events occurred within GIs, which were found to be incompatible in retaining gene order unless these processes were either highly localized to specific sites within the genome, or characterized by symmetrical gene gain and loss (Spring-Pearson et al., 2015). In our study, *bon* gene clusters in the 36 *B. gladioli* strains and *B. cocovenenans* DMSZ11318 demonstrated a conserved gene organization and order. Moreover, some flanking sequences of the *bon* gene cluster insertion region were homologous, which is suggestive of site-specific recombination events involved in the formation of the *bon* gene cluster.

The acquisition of foreign genes can contribute to bacteria’s quick adaptation to a new environment (Bachert et al., 2015). Clustered arrangements of genes are more easily transferred to other species, thus improving the prospects for survival when under selection (Ziemert et al., 2014). The *bon* gene clusters within the 36 *B. gladioli* genomes are so similar to each other indicated that some vertical and horizontal gene transfer events may emerge during this toxin cluster inheritance and spread.

To date, 239 genomes of this species have been published and, of those, *B. cocovenenans* Co14 was the only one which had been tested to be positive for the production of BA. Genomic analysis of the BA biosynthesis gene cluster *bon* of *B. cocovenenans* at species-wide level revealed complex recombination and HGT events contributed to this toxin cluster evolution and formation. Our data also suggests that further sequencing of the *Burkholderia* genome and toxin production testing may lead to a better understanding of the origin and evolution of this lethal foodborne pathovar. These findings can then be used to limit the risk to public health of *B. cocovenenans* food poisoning events in the future. Additionally, as an ANT inhibitor and an ADP/ATP translocator, BA can be used as a pharmacological tool to modulate the properties of the MPTP or ANT in mitochondria, as well as a tool in the elucidation of apoptosis mechanisms. In this respect, a deep understanding for the origin and evolutionary pathway of BA biosynthesis gene cluster would be beneficial for mining other potential biological activities of BA and develop this compound as a drug target candidate.

## Data Availability Statement

The datasets presented in this study can be found in online repositories. The names of the repository/repositories and accession number(s) can be found in the article/Supplementary Material.

## Author Contributions

ZP, JX, and FL contributed to the conceptualization, project administration, and resources. ZP, TD, and YH contributed to the methodology. TD and YH contributed to the software. ML and MB contributed to the validation. ZP, SY, TD, YH, ML, and MB contributed to the formal analysis. ZP contributed to the investigation. ZP and MB contributed to the data curation. ZP, SY, and SF contributed to the writing – original and draft preparation. MB, SF, JX, and FL contributed to the writing – review and editing. YH contributed to the visualization. JX and FL contributed to the supervision. ZP, TD, JX, and FL contributed to the funding acquisition. All authors have made substantial contributions to and have approved the final manuscript.

## Conflict of Interest

The authors declare that the research was conducted in the absence of any commercial or financial relationships that could be construed as a potential conflict of interest.

## Footnotes

1 http://www.broadinstitute.org/software/allpaths-lg/blog/

2 https://www.ncbi.nlm.nih.gov/genome/genomes/3602?

3 https://www.patricbrc.org/

4 https://github.com/ParBLiSS/FastANI

5 https://www.rdocumentation.org/packages/pheatmap/versions/1.0.12/topics/pheatmap

6 https://github.com/sanger-pathogens/Roary

7 https://github.com/sanger-pathogens/Roary/tree/master/contrib/roary_plots

8 https://github.com/sanger-pathogens/snp-sites

9 http://mjsull.github.io/Easyfig/

10 https://antismash.secondarymetabolites.org/

## Supplementary Material

Supplementary Figure 1 Maximum-likelihood phylogenetic tree based on core-genome SNPs of the 239 *Burkholderia gladioli* genomes originating from human clinic, plant, environment, food, and animal samples. The sample sources are shown in solid circles with different colors at the end of each branch. The 36 strains containing the bongkrekic acid biosynthetic gene cluster *bon* are formed three clades and shown in pink background color on the tree. The *B. glumae* ASM993137 genome was used as an outgroup. The tree scale was shown on the left top.

Supplementary Figure 2 Linear alignment and detailed structure of all the 23 complete bongkrekic acid (BA) biosynthetic gene cluster *bon* and its flanking homologous sequences. The gene product names are labeled on the top of *B. cocovenenans* DMSZ11318 *bon* gene cluster. The red arrows represented the ORF*s* in the *bon* gene cluster, the orange arrows represent the flanking sequences, the blue arrows represent non-*bon* genes between the flanking homologous sequences, and the green arrows represent the transposase encoding sequences.

Supplementary Figure 3 Genomic island of *B. cocovenenans* Co14 **(A)**, *B. gladioli* BSR3 **(B)** and *B. gladioli* 3723STDY6437373 **(C)** identified by IslandViewer 4. The bongkrekic acid biosynthetic gene cluster bon regions of *B. cocovenenans* Co14, *B. gladioli* BSR3 and *B. gladioli* 3723STDY6437373 are framed by a blue rectangle.

Supplementary Table 1 The query coverage and nucleotide identity of the complete bongkrekic acid biosynthesis gene clusters to that of *Burkholderia gladioli* pv. *cocovenenans* DMSZ11318 in GenBank.

Supplementary Table 2 The query coverage and nucleotide identity of the complete bongkrekic acid biosynthesis gene clusters to that of *Burkholderia gladioli* pv. *cocovenenans* Co14 in GenBank.

Supplementary Dataset 1 Information of *Burkholderia gladioli* strains and their genomes.

Supplementary Dataset 2 Information of *Burkholderia* spp. strains and their genomes.

Supplementary Dataset 3 Information of 36 BA biosynthesis gene clusters bon containing *Burkholderia gladioli* strains and their genomes.

Supplementary Dataset 4 Comparison of the bongkrekic acid biosynthesis gene clusters of the 36 *bon*-containing *Burkholderia gladioli* genomes.
