# Supplementary figures and images for: Comparative Genomic Analysis of the Foodborne Pathogen Burkholderia gladioli pv. cocovenenans Harboring a Bongkrekic Acid Biosynthesis Gene Cluster
Source: Front Microbiol. 2021 May 17;12:628538. doi: 10.3389/fmicb.2021.628538 (PMC8166232; doi:10.3389/fmicb.2021.628538)

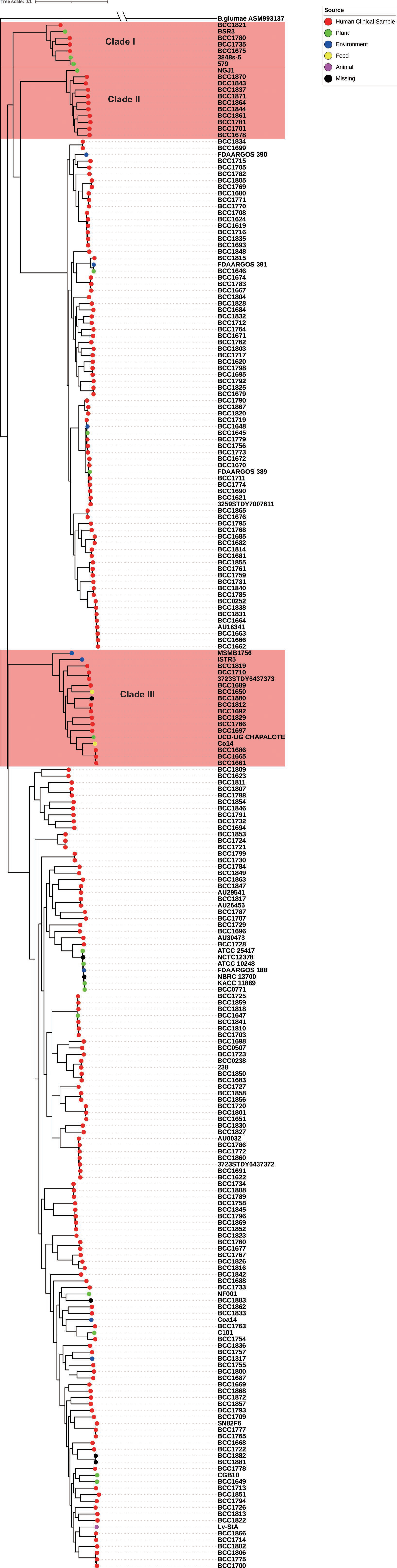

Supplement: Supplementary Figure 1 — Maximum-likelihood phylogenetic tree based on core-genome SNPs of the 239 Burkholderia gladioli genomes originating from human clinic, plant, environment, food, and animal samples. The sample sources are shown in solid circles with different colors at the end of each branch. The 36 strains containing the bongkrekic acid biosynthetic gene cluster bon are formed three clades and shown in pink background color on the tree. The B. glumae ASM993137 genome was used as an outgroup. The tree scale was shown on the left top. [file Image_1.tiff]

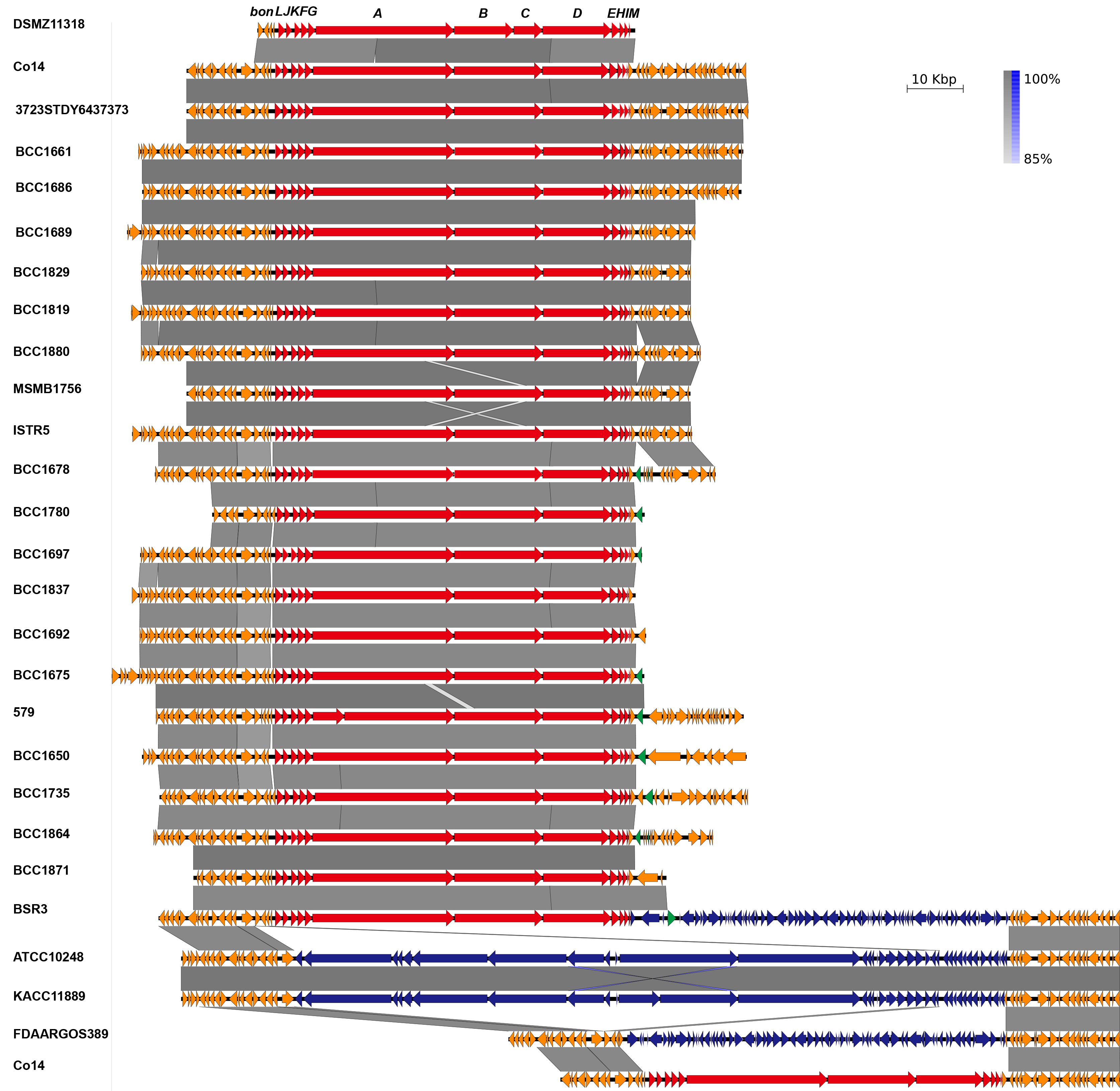

Supplement: Supplementary Figure 2 — Linear alignment and detailed structure of all the 23 complete bongkrekic acid (BA) biosynthetic gene cluster bon and its flanking homologous sequences. The gene product names are labeled on the top of B. cocovenenans DMSZ11318 bon gene cluster. The red arrows represented the ORFs in the bon gene cluster, the orange arrows represent the flanking sequences, the blue arrows represent non-bon genes between the flanking homologous sequences, and the green arrows represent the transposase encoding sequences. [file Image_2.tif]

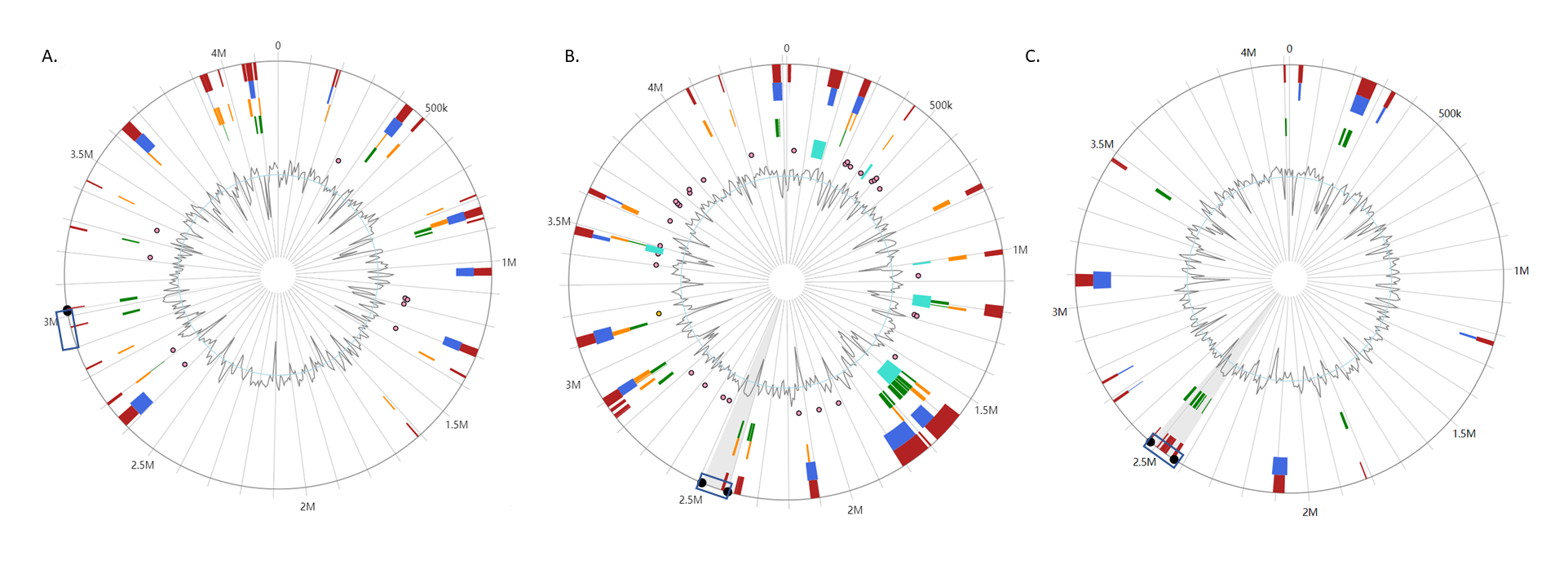

Supplement: Supplementary Figure 3 — Genomic island of B. cocovenenans Co14 (A), B. gladioli BSR3 (B) and B. gladioli 3723STDY6437373 (C) identified by IslandViewer 4. The bongkrekic acid biosynthetic gene cluster bon regions of B. cocovenenans Co14, B. gladioli BSR3 and B. gladioli 3723STDY6437373 are framed by a blue rectangle. [file Image_3.tif]
