# Supplementary material for: Comparative Genomic Analysis of the Foodborne Pathogen Burkholderia gladioli pv. cocovenenans Harboring a Bongkrekic Acid Biosynthesis Gene Cluster
Source: Front Microbiol. 2021 May 17;12:628538. doi: 10.3389/fmicb.2021.628538 (PMC8166232; doi:10.3389/fmicb.2021.628538)
Supplement: Supplementary Table 1 — The query coverage and nucleotide identity of the complete bongkrekic acid biosynthesis gene clusters to that of Burkholderia gladioli pv. cocovenenans DMSZ11318 in GenBank. [file Table_1.docx]

**Supplementary Table 1. The query cover and identity of the complete bongkrekic acid biosynthesis gene clusters to that of *Burkholderia gladioli* pv. *cocovenenans* DMSZ11318 in GeneBank**

| Sequence belongs to the strain | Query cover (%) | Identity (%) |
| --- | --- | --- |
| *B. gladioli* BCC1650 | 100% | 99.95 |
| *B. gladioli* BCC1697 | 100% | 99.52 |
| *B. gladioli* BCC1686 | 100% | 99.42 |
| *B. gladioli* BCC1665 | 100% | 99.42 |
| *B. gladioli* BCC1661 | 100% | 99.42 |
| *B. gladioli* BCC1689 | 100% | 99.41 |
| *B. gladioli* 3723STDY6437373 | 100% | 99.46 |
| *B. gladioli* BCC1710 | 100% | 99.46 |
| *B. gladioli* BCC1880 | 100% | 99.40 |
| *B. gladioli* BCC1819 | 100% | 99.42 |
| *B. gladioli* BCC1692 | 100% | 99.35 |
| *B. gladioli* BCC1812 | 100% | 99.32 |
| *B. gladioli* BCC1829 | 99% | 99.41 |
| *B. gladioli* MSMB1756 | 99% | 99.32 |
| *B. gladioli* ISTR5 | 99% | 99.32 |
| *B. gladioli* BCC1780 | 99% | 98.39 |
| *B. gladioli* BCC1675 | 99% | 98.35 |
| *B. gladioli* BCC1735 | 99% | 98.24 |
| *B. gladioli* 579 | 99% | 98.18 |
| *B. gladioli* BCC1678 | 99% | 98.08 |
| *B. gladioli* BCC1864 | 99% | 98.04 |
| *B. gladioli* BCC1837 | 99% | 98.04 |
| *B. gladioli* BCC1701 | 99% | 97.99 |
| *B. gladioli* BCC1871 | 99% | 97.93 |

*Due to the technical limitation of whole genome sequencing, only the *B. gladioli* genomes containing the intact *bon* cluster were shown in the table.
