# Supplementary material for: Comparative Genomic Analysis of the Foodborne Pathogen Burkholderia gladioli pv. cocovenenans Harboring a Bongkrekic Acid Biosynthesis Gene Cluster
Source: Front Microbiol. 2021 May 17;12:628538. doi: 10.3389/fmicb.2021.628538 (PMC8166232; doi:10.3389/fmicb.2021.628538)
Supplement: Supplementary Table 2 — The query coverage and nucleotide identity of the complete bongkrekic acid biosynthesis gene clusters to that of Burkholderia gladioli pv. cocovenenans Co14 in GenBank. [file Table_2.docx]

**Supplementary Table 2. The query coverage and nucleotide identity of the complete bongkrekic acid biosynthesis gene clusters to that of *Burkholderia gladioli* pv. *cocovenenans* Co14 in GeneBank**

| Sequence belongs to the strain^*^ | Query cover (%) | Identity (%) |
| --- | --- | --- |
| *B. gladioli* BCC1812 | 100 | 99.44 |
| *B. gladioli* BCC1880 | 100 | 99.47 |
| *B. gladioli* BCC1692 | 100 | 99.41 |
| *B. gladioli* BCC1829 | 100 | 99.41 |
| *B. gladioli* MSMB1756 | 100 | 99.39 |
| *B. gladioli* BCC1697 | 100 | 99.38 |
| *B. gladioli* BCC1686 | 100 | 99.33 |
| *B. gladioli* BCC1665 | 100 | 99.33 |
| *B. gladioli* BCC1661 | 100 | 99.33 |
| *B. gladioli* BCC1689 | 100 | 99.31 |
| *B. gladioli* ISTR5 | 100 | 99.39 |
| *B. gladioli* 3723STDY6437373 | 100 | 99.32 |
| *B. gladioli* BCC1710 | 100 | 99.32 |
| *B. gladioli* BCC1650 | 100 | 99.51 |
| *B. gladioli* BCC1819 | 100 | 99.17 |
| *B. cocovenenans* DMSZ11318 | 99 | 99.39 |
| *B. cocovenenans* BSR3 | 99 | 98.46 |
| *B. gladioli* BCC1678 | 99 | 98.20 |
| *B. gladioli* BCC1837 | 99 | 98.18 |
| *B. gladioli* BCC1864 | 99 | 98.14 |
| *B. gladioli* BCC1701 | 99 | 98.12 |
| *B. gladioli* BCC1871 | 99 | 98.06 |
| *B. gladioli* BCC1675 | 99 | 98.36 |
| *B. gladioli* 579 | 99 | 98.05 |
| *B. gladioli* BCC1735 | 99 | 98.38 |
| *B. gladioli* BCC1780 | 99 | 98.16 |

*Due to the technical limitation of whole genome sequencing, only the *B. gladioli* genomes containing the intact *bon* cluster were shown in the table.
